# Role of Endothelial AADC in Cardiac Synthesis of Serotonin and Nitrates Accumulation

**DOI:** 10.1371/journal.pone.0034893

**Published:** 2012-07-19

**Authors:** Charlotte Rouzaud-Laborde, Naïma Hanoun, Ipek Baysal, Jean-Simon Rech, Céline Mias, Denis Calise, Pierre Sicard, Céline Frugier, Marie-Helène Seguelas, Angelo Parini, Nathalie Pizzinat

**Affiliations:** 1 Institut National de la Santé et de la Recherche Médicale, Institut des Maladies Métaboliques et Cardiovasculaires de Rangueil, Toulouse, France; 2 Université Toulouse III, Institut de Médecine Moléculaire de Rangueil, Toulouse, France; 3 Pôle Pharmacie, Centre Hospitalier Universitaire Toulouse, Toulouse, France; 4 Institut National de la Santé et de la Recherche Médicale, Toulouse, France; Goethe University, Germany

## Abstract

Serotonin (5-HT) regulates different cardiac functions by acting directly on cardiomyocytes, fibroblasts and endothelial cells. Today, it is widely accepted that activated platelets represent a major source of 5-HT. In contrast, a supposed production of 5-HT in the heart is still controversial. To address this issue, we investigated the expression and localization of 5-HT synthesizing enzyme tryptophan hydroxylase (TPH) and L-aromatic amino acid decarboxylase (AADC) in the heart. We also evaluated their involvement in cardiac production of 5-HT. TPH1 was weakly expressed in mouse and rat heart and appeared restricted to mast cells. Degranulation of mast cells by compound 48/80 did not modify 5-HT cardiac content in mice. Western blots and immunolabelling experiments showed an abundant expression of AADC in the mouse and rat heart and its co-localization with endothelial cells. Incubation of cardiac homogenate with the AADC substrate (5-hydroxy-L-tryptophan) 5-HTP or intraperitoneal injection of 5-HTP in mice significantly increased cardiac 5-HT. These effects were prevented by the AADC inhibitor benserazide. Finally, 5-HTP administration in mice increased phosphorylation of aortic nitric oxide synthase 3 at Ser (1177) as well as accumulation of nitrates in cardiac tissue. This suggests that the increase in 5-HT production by AADC leads to activation of endothelial and cardiac nitric oxide pathway. These data show that endothelial AADC plays an important role in cardiac synthesis of 5-HT and possibly in 5-HT-dependent regulation of nitric oxide generation.

## Introduction

Serotonin (5-HT) affects a wide range of physiological functions including circadian rhythm, gastrointestinal motility, haemostasis processes and cardiovascular system. Action of 5-HT occurs mainly through activation of its transmembrane receptors expressed by a broad range of cell types in many organs. At the opposite, sites of synthesis of 5-HT appear to be restricted to two principal locations in organism: brain and intestine. In these tissues, 5-HT is synthesized by a two-step enzymatic reaction. The essential amino acid L-tryptophan is first hydroxylated into 5-hydroxy-L-tryptophan (5-HTP) by the limiting enzyme tryptophan hydroxylase. Two isoforms of TPH enzyme (TPH1 and TPH2) have been characterized so far: TPH1 is mainly expressed in the gastrointestinal tract and the pineal gland whereas TPH2 is found in the central nervous system [1]. The 5-HTP generated by TPH enzymes need to be further transformed into 5-HT by L-aromatic amino acid decarboxylase (AADC). AADC is a pyridoxal requiring enzyme abundantly found in all central and peripheral monoaminergic neurons and in intestinal enterochromaffin cells. Part of the 5-HT synthesized in the gastrointestinal tract, is released into the blood stream where it is actively incorporated into dense granules of platelets to maintain low 5-HT concentration in plasma.

Serotonin action regulates different cardiovascular functions [2], [3]. 5-HT released from activated platelets participates in vasoconstriction or vasospasm of coronary arteries during thrombotic events. These effects are mainly produced by activation of 5-HT receptors located on smooth muscle cells which are particularly exposed to blood stream and platelets after atherothrombotic plaque disruption and vessel injury [4]. In physiological situations, 5-HT can produce dilatation of coronary arteries. Indeed, many reports have shown a direct effect of exogenously administrated 5-HT on coronary blood flow in different species (rat, dog, guinea pigs and porcine) where 5-HT or 5-HT agonist dilated coronary arterioles in a dose-dependent manner, both *in vitro* and *in vivo* [5], [6], [7], [8], [9]. This relaxant effect, that appears to be dependent of nitric oxide (NO) release by endothelial cells, is blunted in presence of damaged endothelium or nitric oxide synthase (NOS) inhibitor L-NAME [8], [10], [11], [12]. Serotonin also affects functions of different cells including cardiomyocytes, cardiac fibroblasts and endothelial cells by activation of serotoninergic receptors [13], [14], [15]. However, despite these numerous effects of 5-HT on the heart, only few studies addressed the origin of 5-HT activating these cardiac receptors in physiological situations. Some evidences have previously suggested that 5-HT may be synthesized by the heart itself. Sole et al. observed that cardiac 5-HT levels were significantly reduced after tryptophan hydroxylase inhibitor treatment and remained unchanged after depletion of monoaminergic nerve endings by neurotoxins. This suggests that cardiac 5-HT is not derived from serotoninergic or adrenergic innervations in the heart [16].

In the present work, we sought for expression of 5-HT synthesizing enzyme TPH and AADC in heart and their implication in 5-HT content of cardiac tissue. TPH1 enzyme was restricted to mast cells whereas AADC enzyme co-localized with coronary and microvascular endothelial cells. Participation of AADC in cardiac synthesis of 5-HT *in vivo* was measured by acute pharmacological inhibition of the enzyme activity and injection of 5-HT precursor 5-HTP. This intracardiac synthesis of 5-HT led to an accumulation of nitrates in cardiac tissue by a mechanism that may involve activation of nitric oxide synthase 3 (NOS3).

## Materials and Methods

### Antibodies and Reagents

The anti-TPH1 and anti-α-tubulin antibodies were obtained from Millipore (France). Antibodies directed against AADC and NOS3 proteins were produced from Millipore and Santa Cruz biotechnology (CA, USA), the anti-CD31 antibody from BD Biosciences (Le Pont de Claix, France), and the anti Phospho-NOS3 (Ser1177) from Cell Signaling Technology (Ozyme, France). All chemicals were obtained from Sigma-Aldrich unless otherwise indicated.

### Ethics Statement

*In vivo* studies were conducted in mice and rats under European laws on the protection of animals (86/609/EEC). Mouse experiments were approved and performed according to the guidelines of the Ethics and Animal Safety Committee of INSERM Toulouse/ENVT (agreement number: B315557).

### Animal Experiments

Experimental procedures were carried out in accordance with national law and the European Community guidelines for the use of experimental animals. 129/SvJ mice and Sprague-Dawley rats from Janvier Laboratories (France) aged of 2–4 months were housed in groups under a controlled light/dark cycle and had free access to standard food and tap water. All drugs were dissolved in saline (0.9% NaCl). Mice received one intraperitoneal injection of the peripheral AADC inhibitor benserazide at 100 mg/kg or vehicle 30 min before sacrifice. For combined benserazide and 5-HTP administration, mice received one intraperitoneal injection of 5-HTP at 5 to 20 mg/kg or vehicle 30 min after benserazide (100 mg/kg) or vehicle treatment. Mice were sacrified at different laps of time after the last injection. Degranulation of mast cells was achieved with intraperitoneal injections of compound 48/80 at 1 mg/kg/daily or vehicle for 3 days before sacrifice of mice (supporting information S1) [17]. TPH1−/− mice in a 129/SvJ background were kindly given by Dr Cotté [18].

### 5-HT and 5-HTP Measurements by HPLC

Mice were treated with the peripheral AADC inhibitor benserazide, or 5-HTP or compound H48/80 or saline or a combination of these chemicals as described above. Then, they were anesthetized under isoflurane. Blood from vena cava was collected on heparin as anticoagulant. Mouse hearts were rapidly removed from anesthetized animals, immerged in saline then cut, rinsed once more in saline to eliminate blood, and absorbed in Whatman paper and immediately snap frozen in liquid nitrogen. Parallel experiments were performed by perfusing heart *in situ* with 10 ml of Phosphate Buffer Saline before extraction. Eluted 5-HT and 5HTP were quantified electrochemically (at 0.65 V) and concentrations were calculated in picograms per milligram of organ or micrograms per milliliter of blood and nanograms per milliliter of plasma. Both techniques of heart sampling gave similar quantification of cardiac 5-HT and 5-HTP. Cardiac tissues were weighed and extracted in 5 volumes (vol/wt) of 0.1 N perchloric acid/0.05% disodium EDTA/0.05% sodium metabisulfite as described by Izikki [19]. Ten µl of extracted samples were injected onto a Beckman Ultrasphere 5-µm IP column (Beckman).

### AADC Activity

For concentration-dependent synthesis of 5-HT from 5-HTP, hearts were homogenized in 0.1 M phosphate buffer pH 7.4 supplemented by protease inhibitor cocktail (Roche, France). Eight hundred µg of protein were preincubated at 37°C for 20 min, with 100 µM of AADC cofactor pyridoxal phosphate, 20 µM of monoamine oxidase A inhibitor clorgyline, 0.5 mM ascorbic acid and +/−50 µM benserazide. Then, 10 to 100 µM of 5-HTP were added for 20 min at 37°C. The reaction was stopped by adding 10 µl of perchloric acid 60%. Newly synthesized 5-HT was analyzed by HPLC.

#### RNA extraction and reverse transcription-polymerase chain reaction RT-PCR

Total RNA was isolated from mice heart using Qiagen RNA extraction Kit, DNase treatment was systematically performed according to manufacturer instructions. First-strand cDNA was synthesized from up to 500 ng of total RNA by reverse transcription for 60 minutes at 42°C in a final volume of 20 µl of RT buffer with 100 U of Superscript II (Invitrogen), 0.25 µg random primers, 0.5 mM dNTPs (dATP, dGTP, dCTP, and dTTP), 5 mM dithiothreitol (DTT), 32 U Rnase inhibitor following the manufacturer instructions. Five µl of first strand cDNA was then used to amplify TPH1, TPH2 and AADC fragment by PCR. Reaction mix containing PCR buffer with 1.5 mM MgCl2, 0.2 mM dNTPs, 60 nM of primers (Table 1), 2 U Taq polymerase and reverse transcription reaction was denatured and then amplified by 35 cycles with a DNA thermal cycler (Eppendorff-France). The final extension step was prolonged to 10 min. The absence of contaminants was checked by RT-PCR assays of negative control samples in which the Superscript II was omitted.

**Table 1:** Primer sets used in RT-PCR experiments.

TPH1	F : CCCCTGCTGAAGTCGCAC
	R : TCATACCGCAACTCATTCATGG
TPH2	F : AAGTTGGTGGGCTGGTGAAA
	R : CCGCCAGGAAGTCTCTTGG
AADC	F : CCTCCCCAGAGTTCACACAAG
	R : AAAGAGCGAAATCGTCGCC

**Figure 1:**
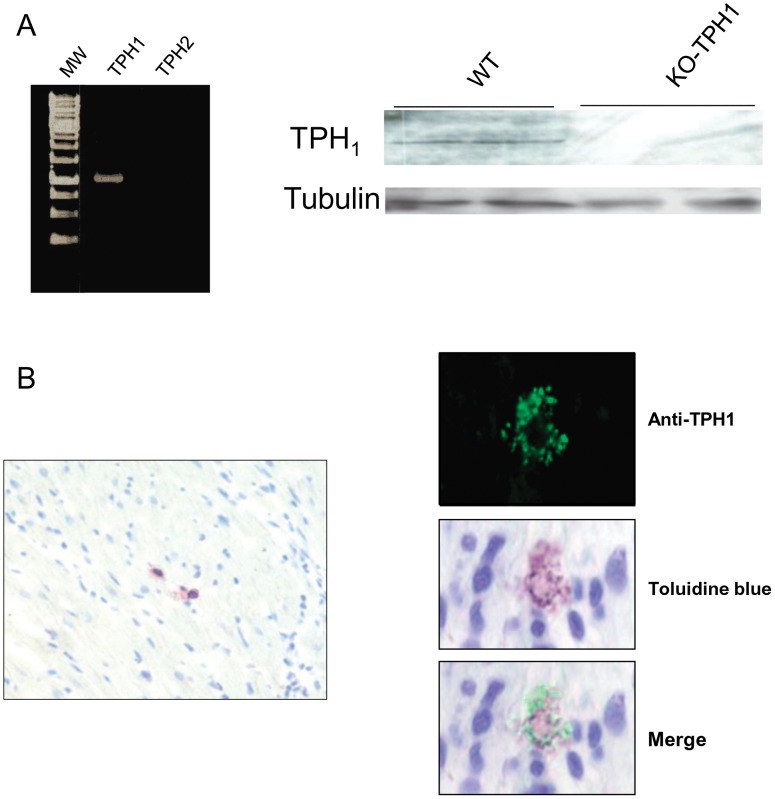
Expression and localization of TPH1 in heart. (A) TPH1 mRNA expression in cardiac tissue was defined by RT-PCR (MW: molecular weight) (left panel) and Western blot (right panel). Immunoblots showed a faint immunoreactive band in heart lysates of 129/SvJ mice but not in those from 129/SvJ TPH1 KO mice. (B) TPH1 immunostaining localized within vesicular granules in rat heart (right and upper panel). Metachromatic granules of mast cells stained with toluidine blue; magnification 400X (right panels). Few mast cells stained with toluidine blue were also present in heart of 129/SvJ mice, magnification 100X (left panel).

**Figure 2:**
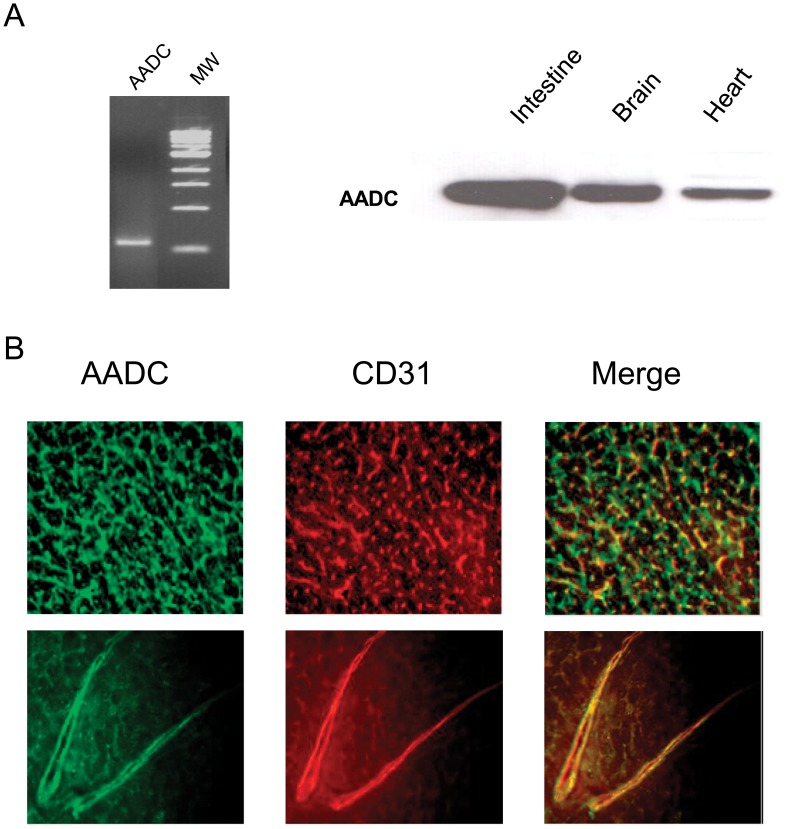
Expression and localization of AADC in cardiac tissue. (A) AADC mRNA expression in mouse cardiac tissue was defined by RT-PCR (left panel) and protein expression by Western blot (right panel) (MW: molecular weight). (B) Immunofluorescent double staining of heart frozen sections with antibodies directed against AADC (a,d green) and CD31 (b,e red). The merged pictures (c, f) show co-localization of AADC and CD31 proteins in microvascular (c) and coronary endothelium (d). Magnification 200X.

### Protein Extraction and Western Blotting

Mouse and rat tissues were lysated using lysis buffer containing 50 mM Tris-HCl, pH 7.5, 150 mM NaCl, 1 mM EDTA, 1 mM EGTA, 1% Triton ×100, 10 µg/ml PMSF, supplemented with complete protease inhibitor cocktail and phosphatase inhibitor cocktail (Roche, France). Proteins were loaded on a 10% polyacrylamide gel and transferred to PVDF membrane. The membrane was blocked with 1% BSA in TBS-Tween 20 (0.1%) (TBST) overnight at 4°C. Anti phospho-NOS3 (1∶500), anti-NOS3 (1∶500), anti-AADC (1∶1000), or anti-TPH1 (1∶500) was used as primary antibody. After incubation with appropriate horseradish peroxidase (HRP) -linked secondary antibody (1∶10,000, 1hour, at room temperature), proteins were detected using the Enhanced Chemo Luminescence reagent reaction (ECL, Millipore, France). Densitometric quantification of Western blot bands was performed using Photoshop 7.0 (Adobe). Protein was measured with a biorad protein assay kit with gamma globulin as a standard.

**Figure 3:**
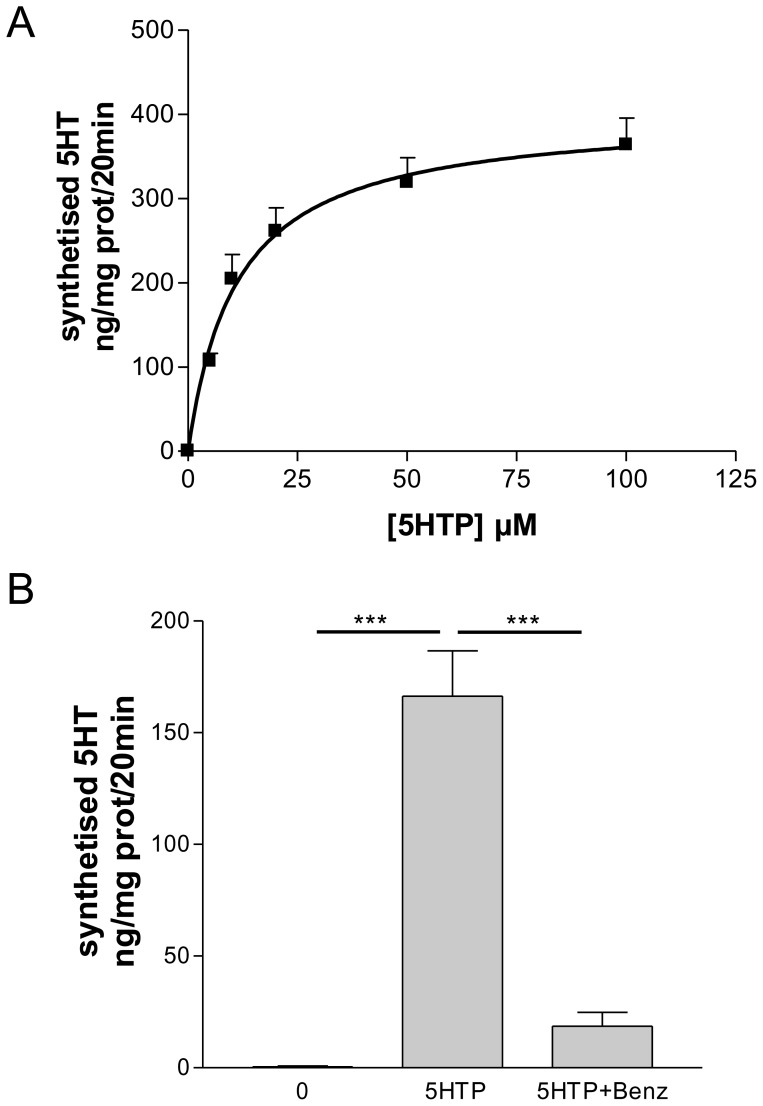
AADC activity in cardiac tissue. (A) Enzyme activity was performed by measuring the conversion of different concentration of 5-hydroxytryptophan (5-HTP) (10–100 µM) into 5-HT in homogenates of mouse cardiac tissue. Values are means ± SEM of 5-HT concentration obtained from three different heart homogenates performed in triplicate. (B) Synthesis of 5-HT from 20 µM 5-HTP in presence or not of 50 µM of AADC inhibitor benserazide. Values are means ± SEM (*n* = 3 to each group) ***P<0.0001.

### Immunostaining

Heart frozen sections were thawed, fixed in 4% paraformaldehyde, washed in PBS, permeabilized in 0.3% Triton X-100 and then incubated in blocking buffer (5% foetal bovine serum in PBS). After that, rabbit anti-AADC Antibody (Ab) (1∶100; Millipore), or goat anti AADC Ab (1∶100; Santa Cruz Biotechnology), or rabbit anti TPH1 Ab (1∶100; Millipore) and the rat anti CD31 Ab (1∶100) were incubated for 1 hour at 37°C. Following PBS washes, sections were treated with FITC-conjugated anti-rabbit Ab (1∶500; Invitrogen) and with Alexa 680-conjugated anti rat antibody (1∶500; Invitrogen), washed again, counterstained with DAPI (Sigma Chemical.), mounted and examined with fluorescent microscope. When needed, sections were counterstained with hematoxylin and toluidine blue (0.5% w/v toluidine blue in 0.5N HCl for 30 min) to identify mast cells in cardiac tissue as described by Gersch et al [20].

**Figure 4:**
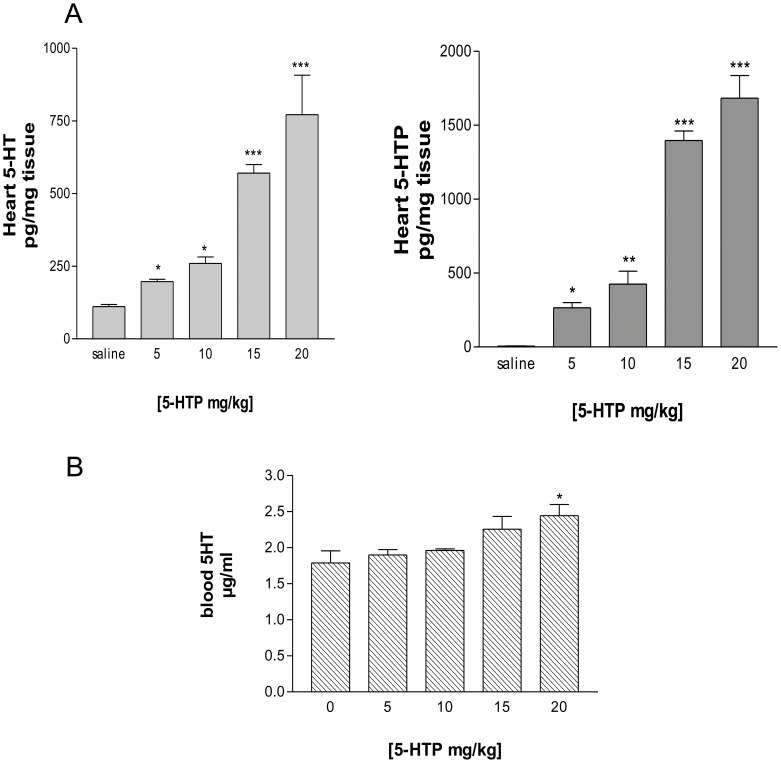
Cardiac and blood 5-HT level after 5-HTP administration. (A) 5-HT and 5-HTP were measured in heart of 129/SvJ mice treated with 5-HTP (5 to 20 mg/kg ip) or saline 30 min before euthanasia. (B) 5-HT concentration in blood of 5-HTP treated mice. Values are means ± SEM (*n* = 3–7 to each group). *P<0.05; **P<0.001; ***P<0.0001 vs. saline.

### NOx Measurement

Mice received intraperitoneal injections of benserazide and/or 5-HTP as explained in animal experiments section. Mice received one intraperitoneal injection of 5-HTP at 20 mg/kg or vehicle 30 min after benserazide (100 mg/kg) or vehicle treatment for combined benserazide and 5-HTP administration. Then, mice were sacrified at different time periods: 5, 10 and 30 minutes. To estimate cardiac level of nitric oxide (NO), the major NO metabolites (nitrates/nitrites) were determined by the Griess reaction in heart homogenates (colorimentric assay kit, Cayman chemical, Europe). The combined oxidation products for NO (nitrites and nitrates) were quantified colorimetrically after reduction with nitrate reductase.

**Figure 5:**
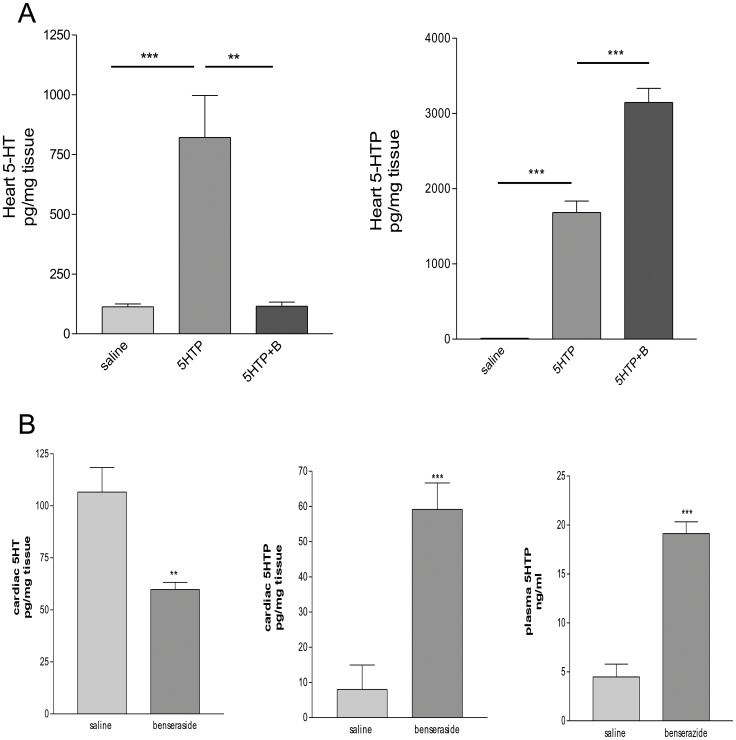
Inhibition of cardiac 5-HT synthesis *in vivo* in presence of the AADC inhibitor benserazide. (A) Mice were pretreated with benserazide (100 mg/kg ip) or saline 30 min before 5-HTP administration (20 mg/kg ip) and they were sacrified 30 min after the last injection. 5-HT and 5-HTP were measured in heart. (B) Mice were treated only with benserazide (100 mg/kg ip) or saline 30 min before euthanasia. 5-HT and 5-HTP were measured in heart and 5-HTP in plasma. Values are means ± SEM (*n* = 8–10 to each group). *P<0.05; **P<0.001 vs. saline.

### Statistical Analysis

All data are reported as mean ± SEM for each group. The significance of differences among different treatment groups was calculated compared using Student’s *t*-test or one-way ANOVA test followed by a post hoc Tukey’s test, as appropriate. A value of *P*<0.05 was considered significant. Enzyme assay curve was plotted using non linear regression with GraphPad prism software.

**Figure 6:**
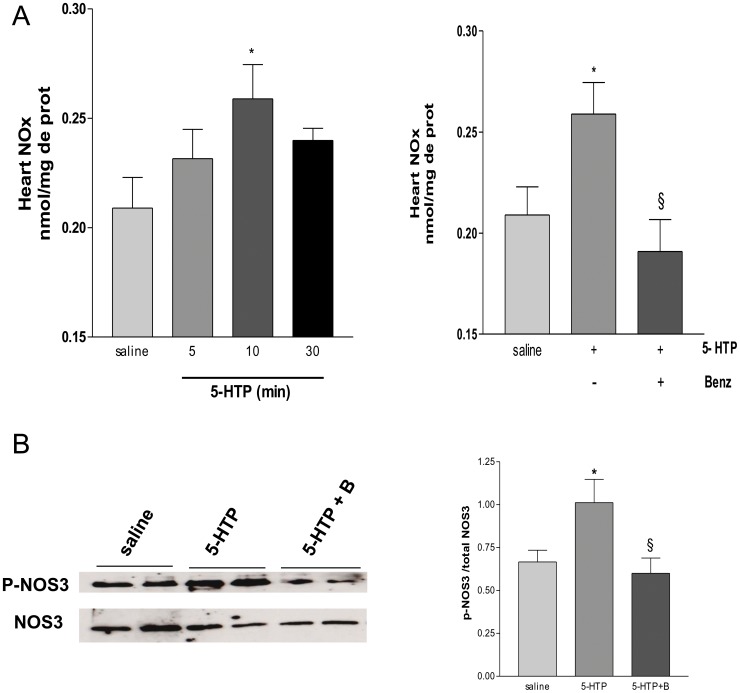
Cardiac NOx content and phosphorylation level of aortic NOS3. (A) Cardiac NOx content in 129/SvJ mice treated with 5-HTP (20 mg/kg ip) or saline 5, 10 or 30 min before euthanasia. A group of mice were pretreated with benserazide (100 mg/kg ip) 30 min before 5-HTP injection. Values are means ± SEM (*n* = 8–10 to each group). *P<0.05 vs. saline, § *P*<0.05 vs. benserazide. (B) Immunodetection of aortic NOS3 phosphorylation 10 min after 5-HTP administration by Western blot. Representative Western blot of aortic endothelial NOS3 phosphorylation levels. Densitometric analysis of Western blot (right panel) (mean ± SEM, *n* = 4 to each group). *P<0.05 vs. saline; § *P*<0.05 vs 5-HTP.

## Results

### Expression and Localization of TPH1 and AADC

Previous studies demonstrated the existence of TPH enzyme in embryonic heart [21]. In order to determine whether TPH is also present in adult mouse heart, we first investigated the TPH1 and TPH2 expression in heart by RT-PCR (fig. 1A left panel). As previously described by Mekontso-Dessap et al., TPH1 mRNA was amplified in mouse heart whereas TPH2 was undetectable [22]. According with these results, Western blot revealed a faint immunostaining for TPH1 in homogenates from *mouse* (fig. 1A right panel) and rat hearts (Fig. S1A) but not from cardiac tissue of TPH1 KO mice (Fig. 1A right panel). In order to further determine the cellular localization of TPH1, we performed immunofluorescence labeling of TPH1 on both *mouse* and rat *hearts.* As shown in Fig. 1B right panel, we observed an intracytoplasmic fluorescent signal inside few cells with the anti-TPH1 antibody in rat heart. This labeling co-localized with metachromatic granules of mast cells stained with toluidine blue (fig. 1B right panel). At the opposite we could not observe any fluorescent signal in mouse cardiac preparations despite, like in rat, few mast cells were detected (fig. 1B left panel).

AADC expression was determined by RT-PCR and Western blot. AADC mRNA was amplified in mice heart (fig. 2A left panel). Immunoblots revealed a 50 kDa immunoreactive protein in mouse (fig. 2A right panel) and rat hearts. Furthermore, expression of the protein was detected in non myocytes fraction *but* not in freshly isolated cardiomyocytes from rat heart suggesting that the enzyme is not ubiquitously present in all cardiac cell types (Fig. S1B).

Immunofluorescence studies were performed to localize the enzyme within the myocardial tissue. AADC green fluorescent immunolabeling co-localized with coronary capillaries and microvascular endothelial cells as shown by double staining with CD31 in fig. 2B. Similar fluorescent staining was obtained with a different source of anti AADC antibody both in mice (not shown) and rat *hearts* (Fig. S1C).

The functionality of AADC expressed in cardiac tissue was determined by enzyme assays. Incubation of the AADC substrate 5-HTP with mouse heart homogenate extracts exhibited a dose dependant accumulation of 5-HT measured by HPLC (fig. 3A). Moreover, this neo-synthesis of 5-HT was prevented in presence of the AADC inhibitor benserazide (fig. 3B).

These results suggest that endothelial AADC may play a major role in cardiac synthesis of 5-HT.

### Role of Endothelial AADC in Cardiac Accumulation of 5-HT *in vivo*

In order to determine whether 5-HT synthesis from its precursor metabolite 5-HTP may occur *in vivo* in the heart by the action of AADC, we first investigated the effects of the AADC substrate administration on cardiac 5-HT levels. With this scope, mice were treated with intraperitoneal injection of 5-HTP at different doses (5 to 20 mg/kg). Mice were sacrificed 30 min after injection and cardiac concentration of 5-HT and 5-HTP were determined. As shown in fig. 4, administration of increasing 5-HTP amounts induced a dose dependent accumulation of cardiac 5-HT (fig. 4A) with only minor changes in blood 5-HT concentration at the highest dose of 5-HTP (20 mg/kg) (fig. 4B). To exclude possible interference of blood platelets with 5-HT determination, experiments were conducted in isolated perfused heart. As observed in vivo, perfusion of heart with 5-HTP generated cardiac 5-HT (Fig. S2).

These results indicate that the increase in cardiac 5-HT is unrelated to modification of platelet derived 5-HT and contamination of cardiac tissue by blood.

The relevance of AADC in the cardiac accumulation of 5-HT was confirmed by experiments using the AADC inhibitor benserazide after 5-HTP administration. Treatment of mice with benserazide, prior to 5-HTP administration, fully prevented the increase in cardiac 5-HT. Moreover, this effect was concomitant to an accumulation of cardiac 5-HTP (fig. 5A).

To further determine the involvement of AADC in the production of cardiac 5-HT in physiological situation, we treated mice only with benserazide, for 30 min before sacrifice. Quantification of 5-HT and its precursor demonstrated that benserazide treatment decreased by about 50% the cardiac 5-HT level and enhanced by about 6-fold and 4-fold the 5-HTP level in heart and plasma respectively (fig. 5B). These results strongly suggest that, in normal condition, plasma 5 HTP serves as substrate for 5-HT cardiac neosynthesis.

Taken together, these results show that endothelial AADC and its substrate 5-HTP play a major role in cardiac production of 5-HT.

### Activation of NOS3 by 5-HTP Treatment

Previous studies showed that 5-HT induces activation of nitric oxide synthase and the following release of nitric oxide (NO) by coronary endothelial cells [7], [15]. Based on these results we next determined whether the conversion of 5-HTP to 5-HT by endothelial AADC may have a functional relevance in nitric oxide generation *in vivo.* In this purpose, we measured the major degradation products of NO nitrite (NO2-) and nitrate (NO3-) following 5-HTP administration. Nitrite/nitrate (NOx) level measured in cardiac ventricles 10 min after 5-HTP injection was significantly higher than that observed in vehicle treated animals (fig. 6A left panel). The increase in cardiac NOx was prevented in mouse treated with benserazide prior to 5-HTP administration (fig. 6A right panel). Interestingly, we found that 5-HTP administration increased the level of phosphorylation at Ser1177 of NOS3 in aorta of mice and this effect was prevented following benserazide administration (fig. 6B). Phosphorylation of this serine, which is located in the reductase domain close to the carboxy-terminus, increases NO synthesis [23]. These results show that endothelial AADC induces the activation of vascular NOS3 and accumulation of cardiac nitrates.

## Discussion

Numerous studies have shown expression of 5-HT receptors in various cardiac cells including cardiomyocytes, fibroblasts, endothelial cells. However the origin of 5-HT interacting with these receptors remains not well understood. To address this issue, we looked for expression and function of 5-HT synthesizing enzymes TPH and AADC in cardiac tissue. We observed that AADC is largely expressed in endothelial cells where it seems to participate in cardiac 5-HT production in physiological conditions. Indeed, pharmacological inhibition of AADC significantly decreased the cardiac level of 5-HT while its precursor 5-HTP accumulated in the tissue. The increase in both cardiac and plasmatic 5-HTP level after benserazide treatment hints that 5-HTP present in plasma probably accumulates in cardiac tissue to be further metabolized by endothelial AADC. These data suggest a dynamic synthesis of 5-HT in the heart. Thus, in physiological situations, stimulation of cardiac serotoninergic receptors may occur independently of platelet activation and degranulation. The endothelial localization of AADC seems to be a common feature of different vascular beds as the enzyme expression and/or its activity have been described in cerebral arteries [24], blood vessel of dental pulp [25] and human pulmonary endothelial cells [26]. At present, the involvement of AADC in 5-HT synthesis in these vascular beds has not been demonstrated. However, it is conceivable that, as we reported in our work, this enzyme may play a role in local synthesis of 5-HT.

Our results showed that the upstream enzyme TPH1 involved in 5-HT synthesis is exclusively localized in mast cells in the adult heart. These results are in agreement with previous observations of TPH expression into intracytoplasmic granules of mast cells in the fetal heart [21]. In addition, we observed that pharmacological degranulation of mast cells with compound 48/80 did not modify the cardiac content of 5-HT (Fig. S3). This suggests that TPH1 in mast cell does not play a significant role in the control of cardiac 5-HT. Ni and coll described the presence of TPH enzyme in peripheral arteries and although we were not able to detect TPH1 expression in other structure than mast cells, the possibility of low levels of TPH1 expression in heart and in coronary arteries cannot be ruled out [27].

5-HT is a well known vasoactive agent that produces either vasodilatation or vasoconstriction effects [1], [28], [29]. These discrepancies observed in 5-HT action are mostly dependent of the presence of functional endothelium. Indeed, the relaxant effect produced by 5-HT, is largely attenuated in the absence of endothelium or after endothelium damage. For instance coronary arterioles isolated from porcine hearts exposed to ischemia/reperfusion presented a significantly reduced 5-HT-dependent dilation [30]. These effects of 5-HT on vascular dilatation appear to be mediated through the release of nitric oxide. Indeed, Ellwood and coll. showed that 5-HT dilates guinea pig coronary arteries largely by the release of NO from the coronary endothelium [7]. Production of nitric oxide by coronary artery endothelial cells after serotoninergic stimulation was also confirmed *in vitro* in human and was dependent of serotoninergic receptors expressed by these cells [15].

We found that 5-HTP injection in vivo produced an increase in tissue levels of the nitric oxide metabolites nitrite/nitrate. This modification of NOx level was also observed ex vivo after 5-HTP perfusion in isolated hearts (Fig. S2). This suggests that, in the heart, AADC dependant 5-HT production activates nitric oxide pathway. Although, phosphorylation of cardiac NOS3 was too low to be detected in our experimental conditions, we could measure an increase in phosphorylation at Ser1177 of aortic NOS3 following 5-HTP treatment. This phosphorylation of Ser 1177 residue is known to be critical for eNOS activation and was previously observed *in vitro* after serotoninergic receptors stimulation [31]. These results suggest that the increase of 5-HT synthesis by endothelial AADC may have functional relevance especially through the activation of nitric oxide pathway in the arteries and the heart.

In conclusion, our results showed that endothelial AADC and availability of 5-HTP represent key factors responsible for cardiac synthesis of 5-HT that could regulate 5-HT mediated effects on different cardiac and vascular cells. These results open new perspectives in the comprehension of mechanisms controlling cardiac production of 5-HT and their potential involvement in physiopathological situations.

## Supporting Information

Figure S1 **TPH1 and AADC expression in rat heart.** Rat tissue extracts (40 µg of intestine and 80 µg of heart) were obtained as described in “Materials and Methods” and analyzed by Western blot for TPH1 expression (A). 40 µg of rat tissue extract (intestine, brain and heart) were analyzed by Western blot for and AADC expression (B). Immunofluorescent staining of AADC in rat cardiac tissue was performed as described in “Materials and Methods”. Anti AADC antibody used was sc-46909 (Santa Cruz) (left panel) and DAPI was used to stain nuclei (right panel). Arrows indicate vascular positive staining (C). Magnification 200X.
(TIF)

Figure S2 **AADC activity and Nox level in perfused heart.** 5HT determination (A) and NOx content (B) in isolated heart perfused with 25 µM 5-HTP for 20 min at 37°C. For inhibition of AADC, hearts were pretreated with 100 µM benserazide for 10 min before 5-HTP perfusion. Values are means ± SEM (n = 3–6 to each group). *P<0.05 vs. saline, § P<0.05 vs. benserazide.
(TIF)

Figure S3 **5-HT concentration in heart and blood after treatment of mice with compound 48/80.** Cardiac and blood 5HT levels after treatment of 129SVJ mice with the mast cell stimulator compound 48/80 at the dose of 1 mg/kg for 3 days. Values are means ± SEM (n = 4 to each group). ns: not significant.
(TIF)

Supporting Information S1 **Supplemental data.**
(DOC)

## References

[1] Watts SW (2009). The love of a lifetime: 5-HT in the cardiovascular system.. Am J Physiol Regul Integr Comp Physiol.

[2] Kaumann AJ, Levy FO (2006). 5-hydroxytryptamine receptors in the human cardiovascular system.. Pharmacol Ther.

[3] Villalon CM, Centurion D (2007). Cardiovascular responses produced by 5-hydroxytriptamine:a pharmacological update on the receptors/mechanisms involved and therapeutic implications.. Naunyn Schmiedebergs Arch Pharmacol.

[4] Takano S, Hoshino Y, Li L, Matsuoka I, Ono T (2004). Dual roles of 5-hydroxytryptamine in ischemia-reperfusion injury in isolated rat hearts.. J Cardiovasc Pharmacol Ther.

[5] Bouchard JF, Chouinard J, Lamontagne D (2000). Participation of prostaglandin E2 in the endothelial protective effect of ischaemic preconditioning in isolated rat heart.. Cardiovasc Res.

[6] DeFily DV, Kuo L, Chilian WM (1996). PAF attenuates endothelium-dependent coronary arteriolar vasodilation.. Am J Physiol 270(6 Pt.

[7] Ellwood AJ, Curtis MJ (1996). Mechanism of 5-hydroxytryptamine-induced coronary vasodilation assessed by direct detection of nitric oxide production in guinea-pig isolated heart.. Br J Pharmacol.

[8] Wang W, Hein TW, Zhang C, Zawieja DC, Liao JC (2011). Oxidized low-density lipoprotein inhibits nitric oxide-mediated coronary arteriolar dilation by up-regulating endothelial arginase I. Microcirculation.

[9] Marcus ML, Chilian WM, Kanatsuka H, Dellsperger KC, Eastham CL (1990). Understanding the coronary circulation through studies at the microvascular level.. Circulation.

[10] Zhang C, Hein TW, Wang W, Chang CI, Kuo L (2001). Constitutive expression of arginase in microvascular endothelial cells counteracts nitric oxide-mediated vasodilatory function.. Faseb J.

[11] Tiefenbacher CP, Chilian WM, Mitchell M, DeFily DV (1996). Restoration of endothelium-dependent vasodilation after reperfusion injury by tetrahydrobiopterin.. Circulation.

[12] Qamirani E, Ren Y, Kuo L, Hein TW (2005). C-reactive protein inhibits endothelium-dependent NO-mediated dilation in coronary arterioles by activating p38 kinase and NAD(P)H oxidase.. Arterioscler Thromb Vasc Biol.

[13] Yabanoglu S, Akkiki M, Seguelas MH, Mialet-Perez J, Parini A (2009). Platelet derived serotonin drives the activation of rat cardiac fibroblasts by 5-HT2A receptors.. J Mol Cell Cardiol.

[14] Villeneuve C, Caudrillier A, Ordener C, Pizzinat N, Parini A (2009). Dose-dependent activation of distinct hypertrophic pathways by serotonin in cardiac cells.. Am J Physiol Heart Circ Physiol.

[15] Ishida T, Kawashima S, Hirata K, Yokoyama M (1998). Nitric oxide is produced via 5-HT1B and 5-HT2B receptor activation in human coronary artery endothelial cells.. Kobe J Med Sci.

[16] Sole MJ, Shum A, Van Loon GR (1979). Serotonin metabolism in the normal and failing hamster heart.. Circ Res.

[17] Wei JF, Wei XL, Mo YZ, He SH (2009). Induction of mast cell accumulation, histamine release and skin edema by N49 phospholipase A2.. BMC Immunol.

[18] Cote F, Thevenot E, Fligny C, Fromes Y, Darmon M (2003). Disruption of the nonneuronal tph1 gene demonstrates the importance of peripheral serotonin in cardiac function.. Proc Natl Acad Sci U S A.

[19] Izikki M, Hanoun N, Marcos E, Savale L, Barlier-Mur AM (2007). Tryptophan hydroxylase 1 knockout and tryptophan hydroxylase 2 polymorphism: effects on hypoxic pulmonary hypertension in mice.. Am J Physiol Lung Cell Mol Physiol.

[20] Gersch C, Dewald O, Zoerlein M, Michael LH, Entman ML (2002). Mast cells and macrophages in normal C57/BL/6 mice.. Histochem Cell Biol.

[21] Manjarrez-Gutierrez G, Camacho-Calderon N, Mercado-Camargo R, Boyzo-Montes de Oca A, Arvizu-Flores A (2009). Characterization of serotonergic cells in fetal heart tissue.. Cir Cir.

[22] Mekontso-Dessap A, Brouri F, Pascal O, Lechat P, Hanoun N (2006). Deficiency of the 5-hydroxytryptamine transporter gene leads to cardiac fibrosis and valvulopathy in mice.. Circulation.

[23] Mount PF, Kemp BE, Power DA (2007). Regulation of endothelial and myocardial NO synthesis by multi-site eNOS phosphorylation.. J Mol Cell Cardiol.

[24] Lopez de Pablo AL, Moreno MJ, Marco EJ (1996). In vivo tryptophan hydroxylase activity in rat major cerebral arteries is decreased by dorsal raphe nucleus lesions.. J Neurochem.

[25] Nomura T, Inoue K, Creveling CR, Komatsu F, Ohta N (1996). Immunocytochemical localization of aromatic L-amino acid decarboxylase and catechol-O-methyltransferase in blood vessel wall of the human dental pulp.. Brain Res.

[26] Eddahibi S, Guignabert C, Barlier-Mur AM, Dewachter L, Fadel E (2006). Cross talk between endothelial and smooth muscle cells in pulmonary hypertension: critical role for serotonin-induced smooth muscle hyperplasia.. Circulation.

[27] Ni W, Geddes TJ, Priestley JR, Szasz T, Kuhn DM (2008). The existence of a local 5-hydroxytryptaminergic system in peripheral arteries.. Br J Pharmacol.

[28] Houston DS, Vanhoutte PM (1988). Comparison of serotonergic receptor subtypes on the smooth muscle and endothelium of the canine coronary artery.. J Pharmacol Exp Ther.

[29] Cocks TM, Arnold PJ (1992). 5-Hydroxytryptamine (5-HT) mediates potent relaxation in the sheep isolated pulmonary vein via activation of 5-HT4 receptors.. Br J Pharmacol.

[30] Hein TW, Zhang C, Wang W, Chang CI, Thengchaisri N (2003). Ischemia-reperfusion selectively impairs nitric oxide-mediated dilation in coronary arterioles: counteracting role of arginase.. Faseb J.

[31] Asada M, Ebihara S, Yamanda S, Niu K, Okazaki T (2009). Depletion of serotonin and selective inhibition of 2B receptor suppressed tumor angiogenesis by inhibiting endothelial nitric oxide synthase and extracellular signal-regulated kinase 1/2 phosphorylation.. Neoplasia.

