# Supplementary figures and images for: Role of Endothelial AADC in Cardiac Synthesis of Serotonin and Nitrates Accumulation
Source: PLoS One. 2012 Jul 19;7(7):e34893. doi: 10.1371/journal.pone.0034893 (PMC3400593; doi:10.1371/journal.pone.0034893)

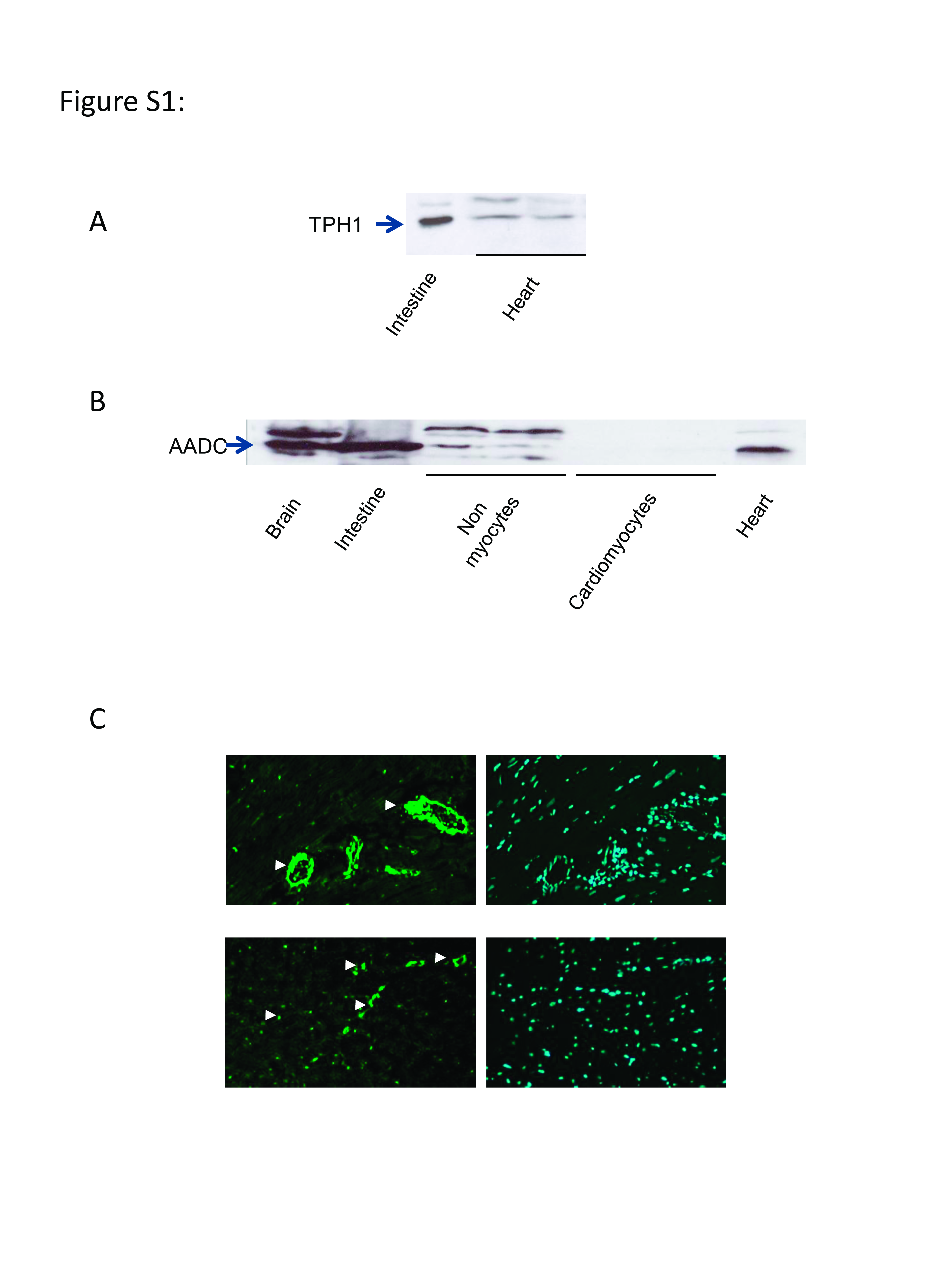

Supplement: Figure S1 — TPH1 and AADC expression in rat heart. Rat tissue extracts (40 µg of intestine and 80 µg of heart) were obtained as described in “Materials and Methods” and analyzed by Western blot for TPH1 expression (A). 40 µg of rat tissue extract (intestine, brain and heart) were analyzed by Western blot for and AADC expression (B). Immunofluorescent staining of AADC in rat cardiac tissue was performed as described in “Materials and Methods”. Anti AADC antibody used was sc-46909 (Santa Cruz) (left panel) and DAPI was used to stain nuclei (right panel). Arrows indicate vascular positive staining (C). Magnification 200X. (TIF) [file pone.0034893.s001.tif]
