# Supplementary material for: Role of Endothelial AADC in Cardiac Synthesis of Serotonin and Nitrates Accumulation
Source: PLoS One. 2012 Jul 19;7(7):e34893. doi: 10.1371/journal.pone.0034893 (PMC3400593; doi:10.1371/journal.pone.0034893)
Supplement: Supporting Information S1 — Supplemental data. (DOC) [file pone.0034893.s004.doc]

Supporting Information S1. Supplemental data

Cardiomyocytes were isolated from Sprague-Dawley rats, using a protocol based on previously described procedures [1]. Rats were handled in accordance with the procedures outlined in Council Directive 86/609/EEC. Rats were anesthetized with 45 mg/kg ip pentobarbital, and the heart was rapidly excised, mounted in a Langendorff apparatus, and perfused for 20 min with low-calcium solution (LCS) prewarmed at 37°C [117 mM NaCl, 5.7 mM KCl, 4.4 mM NaHCO_3_, 1.5 mM KH_2_PO_4_, 1.7 mM MgCl_2_, 11.7 mM d(+)-glucose, 21 mM HEPES, 20 mM taurine, 10 mm creatine (pH 7.2)]. The solution was then quickly changed to LCS plus 1 mg/ml collagenase type I, 0.03 mg/ml dispase (Worthington Biochemicals), and 1 mg/ml albumin for 10 min. The heart was minced, and the pieces were stirred in LCS. Cardiomyocytes present in the supernatant were purified by gravity sedimentation, collected, and snap frozen in liquid nitrogen. Non myocytes fraction was isolated from the ventricles of adult Sprague–Dawlay rats. Ventricles were minced and digested in 0.05% collagenase (Serva, Heidelberg, Germany) containing solution at 37 °C. Cells were pre-plated for 3 h on primaria cell culture dish during which non myocyte cells adhered to the dish. After pre-plating, the medium was removed and the attached cells were maintained until confluence in DMEM/F12 containing 10% fetal bovine serum and penicillin streptomycin (Invitrogen) at 37 °C and 5% CO_2_. [2].

Isolated heart preparation

Male 129sVL mice were anaesthetized with pentobarbital (300 mg/kg) and heparin (150 U) intraperitoneally. The chest was immediately opened and the heart rapidly excised and cannulated through the aorta on Langendorff apparatus. Perfusion was started immediately with Krebs solution (130 mM NaCl, 5.4 mM KCl, 0.4 mM NaH2PO4, 1.4 mM MgCl2, 10mM glucose, 4.2 mM HEPES, 20 mM taurine, 10 mm creatine (pH 7.3) supplemented with 100µM pyridoxal phosphate, 20 µM of clorgyline, 0.5 mM ascorbic acid and +/- 50µM benserazide for 10min. Then, hearts were perfused with Krebs solution in the presence of 25 µM of 5-HTP for an additional 20 min at 37°C. Hearts were removed and snap frozen in liquid nitrogen.

Degranulation of mast cells was achieved with intraperitoneal injections of compound 48/80 at 1 mg/kg/daily or vehicle for 3 days before sacrifice of mice. Then, blood from vena cava was collected on heparin as anticoagulant from isoflurane anesthezised mice. Mouse hearts were rapidly removed, immerged in saline then cut, rinsed once more in saline to eliminate blood, and absorbed in Whatman paper and immediately snap frozen in liquid nitrogen.

Bibliography:

_1. Villeneuve, C., et al._, *Dose-dependent activation of distinct hypertrophic pathways by serotonin in cardiac cells.* Am J Physiol Heart Circ Physiol, 2009. **_297_**_(2): p. H821-8_.

_2. He, Q. and M.J. Spiro_, *Isolation of rat heart endothelial cells and pericytes: evaluation of their role in the formation of extracellular matrix components.* J Mol Cell Cardiol, 1995. **_27_**_(5): p. 1173-83_.
